# Towards specialized dementia risk reduction services for those with first cognitive symptoms: A mixed-method study into risk awareness, needs, and preferences among individuals with subjective cognitive decline and mild cognitive impairment from memory clinic and community settings and memory clinic professionals

**DOI:** 10.1177/13872877261440958

**Published:** 2026-04-17

**Authors:** Lotte S. Truin, Irene S. Heger, Sophie C. P. M. Wimmers, Kay Deckers, Marjolein E. de Vugt, Sebastian Köhler

**Affiliations:** 1 Alzheimer Centre Limburg, Department of Psychiatry and Neuropsychology, Mental Health and Neuroscience Research Institute (MHeNs), 5211 Maastricht University, Maastricht, The Netherlands

**Keywords:** Alzheimer's disease, lifestyle, mild cognitive impairment, prevention, risk factors, subjective cognitive decline

## Abstract

**Background:**

Individuals with subjective cognitive decline (SCD) and mild cognitive impairment (MCI) are at increased risk of dementia and may benefit from lifestyle-based strategies for secondary prevention in clinical practice.

**Objective:**

To examine risk awareness, needs and preferences for dementia risk reduction in individuals with self-reported SCD and MCI and memory clinic professionals.

**Methods:**

Using a mixed-methods approach, we conducted online surveys to examine risk awareness, needs and preferences, and semi-structured interviews to explore barriers and facilitators related to online dementia risk reduction.

**Results:**

1167 individuals with SCD (n = 1092) and MCI (n = 75), predominantly community-based research volunteers and a smaller group recruited via memory clinics, and 58 professionals completed the survey. Among SCD/MCI individuals, 39.5% were unaware that dementia risk can be reduced through lifestyle changes, 97.5% were interested in obtaining such information, and 97.9% considered using an online dementia risk management tool. Among professionals, 10.3% were unaware of dementia risk reduction, and 87.0% considered using a tool on this topic during consultations. Cardiovascular risk factors were poorly recognized by SCD/MCI individuals and, though acknowledged by professionals, rarely discussed in consultations. Interviews with SCD/MCI individuals (n = 14) and professionals (n = 9) highlighted several areas supporting implementation of an online dementia risk management tool (e.g., personalization, up-to-date information, coaching).

**Conclusions:**

Dementia risk awareness was low among SCD/MCI individuals, and high among memory clinic professionals. Both groups showed strong interest in using an online dementia risk management tool, and specific content, design and delivery features may influence engagement. Findings should be interpreted recognizing the mostly online-recruited, higher-educated study sample.

## Introduction

A substantial proportion of dementia cases worldwide are attributable to modifiable risk factors, including physical inactivity, smoking, and social isolation.^1^ Therefore, prioritizing lifestyle-based risk reduction strategies for dementia is essential alongside research into pharmacological treatments. By positioning these approaches as complementary and equally necessary, a more comprehensive strategy can be developed to reduce dementia incidence in the future. Individuals with subjective cognitive decline (SCD) and mild cognitive impairment (MCI) are at increased risk of dementia and are, therefore, key populations for dementia risk reduction interventions.^2–4^ SCD refers to self-reported cognitive decline without measurable deficits, while MCI involves objective impairment in at least one cognitive domain without significant interference in daily life.^2^

Targeting risk awareness and increasing knowledge are crucial first steps in facilitating lifestyle modifications promoted by preventive interventions.^5–7^ However, much of our current understanding of dementia risk awareness comes from studies involving the general population, which show that a large portion of people are still unaware of the dementia risk reduction possibilities.^8–13^ It is essential to understand the specific risk awareness, and information needs and barriers, of individuals with SCD and MCI to develop appropriate interventions that adequately inform and empower them to take preventive action.

In high-income countries, individuals with SCD and MCI are often referred to memory clinics for further diagnosis and support, providing an opportunity to implement strategies for the secondary prevention of dementia through the involvement of healthcare professionals.^14^ Through tailored communication and risk counselling, they can assist individuals with SCD and MCI in understanding the impact of modifiable dementia risk factors. Incorporating dementia risk reduction advice into routine clinical care may not only enhance risk awareness but could also act as a motivational trigger for actual lifestyle changes.^15^ A study with 160 memory clinic professionals from 21 European countries reported that professionals expressed interest in practical support for clinician-patient communication that enhances patient understanding and engagement.^16^ This may further indicate a need among memory clinic professionals for additional support in communicating about dementia risk reduction within the memory clinic setting. While we are not aware of any evidence-based interventions currently available to address this need, it is possible that some forms of support exist locally or informally. Thus, it is also important to explore memory clinic professionals’ risk awareness, needs, and barriers to effective communication about dementia risk reduction, particularly in high-risk populations. These insights will support efforts to equip memory clinic professionals with appropriate evidence-based resources for dementia risk reduction communication, which is in line with current recommendations.^14,17,18^

Therefore, the aim of this mixed-method study was to 1) explore awareness of dementia risk; and 2) understand the needs and preferences regarding dementia risk reduction communication among individuals with SCD and MCI and memory clinic professionals. These findings could further guide the development of targeted resources aimed at improving dementia risk reduction knowledge and communication in the memory clinic setting.

## Methods

### Study design and data integration

This study integrated quantitative and qualitative data to obtain a comprehensive understanding of risk awareness, needs, and preferences for dementia risk reduction among individuals with SCD and MCI, as well as memory clinic professionals. Specifically, we used an explanatory sequential mixed-methods design.^19,20^ The “connecting,” “building,” and “merging” approaches^19^ were applied at different stages of data integration: during sampling, data collection, and interpretation, respectively. First, participants for the semi-structured, in-depth interviews were recruited from the pool of participants who responded to our online survey (connecting). Second, results from the survey partly informed the development of the interview topic guide (building). For example, preferences regarding the format for receiving dementia risk reduction information (paper materials, online, or a combination) were further explored in in-depth interviews by asking participants to elaborate on their choices. Third, we combined the qualitative and quantitative data during analysis to generate comprehensive, integrated insights (merging). The study protocol was approved by the Medical Ethics Committee of Maastricht University Medical Centre+ (#2022-3252), The Netherlands.

### Participants and recruitment

We included Dutch-speaking individuals with clinician-referred or self-reported SCD and MCI, as well as memory clinic professionals. Participants with SCD and MCI were initially recruited for the online survey through two pipelines: 1) memory clinic professionals within the Dutch Memory Clinic Network,^21^ and 2) the online Dutch Brain Research Registry, which focusses predominantly on community-based research volunteers rather than memory clinic patients and serves as a public gateway to scientific studies on brain disorders.^22^ Memory clinic professionals were recruited for the survey via announcements on the Dutch Memory Clinic Network website, newsletter, social media, and at the 7th Dutch Memory Clinic Network conference, as well as through the Alzheimer Centre Limburg's website and social media channels. Participants for the in-depth interviews were subsequently recruited from those who had already completed the online survey, indicated interest in a follow-up interview, and provided consent to be contacted for this purpose. For participants with SCD and MCI, eligibility was restricted to individuals who reported at least one visit to a memory clinic. From this pool, a random sample of 40 individuals with SCD and MCI was invited by email by the research team. All memory clinic professionals (N = 19) who expressed interest were similarly invited by email. Invitees who consented to participate were included in the final interview sample. Online informed consent was obtained prior to survey participation, and written informed consent was obtained before interview participation.

### Survey

From September 2022 to April 2023, participants completed our survey via Qualtrics XM, providing demographic details such as age, gender, and education (low/intermediate/high, based on the Dutch educational system). Those with SCD and MCI answered additional questions about marital status, perceived health (poor to excellent), frequency of memory clinic visits, and diagnosis history (SCD, MCI, dementia). Participants were classified as having SCD if they (1) reported cognitive complaints, and (2) indicated that they had never visited a memory clinic or selected one of the following reasons for attending a memory clinic: “I have complaints about my thinking abilities, but I don’t know (yet) what is going on”, “I have complaints about my thinking abilities, but no cognitive disorder was diagnosed”, or “I don’t know why I went to the memory clinic”. Participants were classified as having MCI if they selected “A mild cognitive disorder has been diagnosed”. For memory clinic professionals, we collected their profession (psychologist, physician/specialist, nurse/nurse practitioner, other), years of experience, employment duration in a memory clinic, and the hospital type (general or academic). All participants were asked about their knowledge of dementia (none, limited, fair, good, excellent). Dementia risk awareness was assessed using survey items that originate from an awareness survey which has been employed in several other studies.^8–13^ Specifically, participants were asked to rate the statement ‘There is nothing you can do to lower your risk of dementia’ on a 5-point Likert scale, ranging from ‘strongly disagree’ to ‘strongly agree’. Responses were dichotomized into ‘aware’ and ‘unaware’; with “strongly disagree” and “disagree” classified as aware, and “neither agree nor disagree”, “agree”, and “strongly agree” classified as unaware. Knowledge of fifteen individual modifiable dementia risk and protective factors^23^ was assessed similarly, with participants indicating on the same 5-point Likert scale the extent to which they agreed that these factors could increase dementia risk (e.g., ‘Regular physical activity lowers the risk of dementia’). All statements regarding individual factors were formulated correctly, indicating that agreement with the statement signifies knowledge of that factor. Items related to the needs and preferences of participants with SCD and MCI focused on their interest in receiving information on dementia risk reduction, preferred methods of delivery (paper materials, online, combination), perceived barriers for applying this information (insufficient knowledge, insufficient time, financial problems, insufficient motivation, difficult to arrange, health issues, other), and their willingness to use an online tool (free app or website) for personalized dementia risk reduction, as such tools also exist for people without memory complaints.^9,24,25^ For memory clinic professionals, these items focused on current practices on dementia risk reduction (e.g., what risk and protective factors are discussed and personalized information for patients), their perceived need for a tool to support memory clinic patients, and their likelihood of using such a tool, including potential reasons for or against its use. Items on needs and preferences were based on previous findings from the literature.^8,16,26^ The complete translated surveys can be found in Supplemental Material 1.

### Semi-structured interviews

Between December 2022 and April 2023, we conducted in-depth interviews to further explore the needs and preferences on dementia risk reduction, and potential barriers and facilitators for using an online dementia risk reduction tool. We invited those participants with SCD and MCI who had visited a memory clinic, and they were asked about their interest in receiving information on brain health and lifestyle, preferred content formats (e.g., digital or non-digital), personalization options, and content length. Memory clinic professionals were asked to provide their views on the design and usefulness of a hypothetical tool in practice and its role in facilitating discussions on lifestyle and brain health. Both participants with SCD and MCI and memory clinic professionals discussed terminology preferences (e.g., brain health versus dementia risk), device options for accessing the tool, and the potential role of coaching to support tool usage. All participants also reflected on factors that could facilitate or hinder tool engagement, with memory clinic professionals specifically highlighting aspects that might improve patient adherence. The topic lists for the semi-structured interviews were developed based on findings from our online survey and previous similar studies.^26,27^ The complete translated topic lists can be found in Supplemental Material 2. The interviews lasted approximately 1 to 1.5 h. Each interview was conducted by a primary interviewer (L.S.T.) while a second researcher (I.S.H.) observed, took notes, and posed additional questions as needed to address topics that may have been overlooked by the primary interviewer. Interviews were conducted either in-person or online via Zoom, depending on the participant's preference. An iterative approach was applied, with preliminary findings from earlier interviews informing and refining the focus of subsequent ones. Data collection continued until data saturation was achieved, meaning no new themes emerged, and strong repetition was observed across interviews.^28^ All interviews were audio-recorded and transcribed verbatim.

### Data analysis

Descriptive statistics were used to summarize participant characteristics, and responses to questions on risk awareness, needs, and preferences related to dementia risk reduction. χ^2^-tests (or Fisher's exact tests in case of expected cell frequency below five in any cell) were used to examine potential differences in these outcomes by age group (< 65 years, ≥65 years), gender (female, male, other), education level (low, intermediate, high), diagnosis (SCD, MCI), recruitment source (Dutch Memory Clinic Network, Dutch Brain Research Registry), prior visit to a memory clinic (yes, no or unsure) and self-perceived dementia knowledge (excellent/good/considerable, limited/poor) for participants with SCD and MCI. Among memory clinic professionals, χ^2^-tests (or Fisher's exact tests in case of expected cell frequency below five in any cell) assessed differences by sex (female, male, other), education level (low, intermediate, high), profession (psychologist, physician/specialist, nurse/nurse practitioner, other), and memory clinic type they worked (general hospital, academic hospital, other). Quantitative analyses were done in Stata 17.0 (StataCorp, College Station, TX, USA) using two-sided tests at an alpha level of 0.05.

Thematic content analysis was conducted on the interview transcripts using ATLAS.ti version 24. A six-phase approach was used, following established recommendations for thematic analysis.^29^ First, two researchers (L.S.T. and S.C.P.M.W.) familiarized themselves with the data by reading all transcripts. Second, transcripts were independently coded, using an open coding approach that allowed codes to emerge inductively from the data. Third, codes were compared between researchers L.S.T. and S.C.P.M.W., and discrepancies were resolved through discussion with researcher I.S.H. until consensus was reached. From this process, initial thematic categories were identified. Fourth, thematic categories were discussed in depth with the other authors. Fifth, main and subthemes were defined. Finally, the authors discussed and verified the themes in a final meeting.

## Results

### Survey of individuals with SCD and MCI

Participants who reported having dementia (n = 5) and who did not answer a question related to dementia risk reduction (n = 198) were excluded, yielding a total of 1167 individuals with SCD (n = 1092) and MCI (n = 75) for further study. Most individuals with SCD and MCI were recruited via the Dutch Brain Research Registry (95.5%) and 20.3% had previously visited a memory clinic (see Table 1 for further demographics). All proportions are reported based on the number of respondents per item; item-level missing data were low (≤4.3%) and are therefore not shown separately.

**Table 1.** Demographics of survey individuals with SCD (n = 1092) and MCI (n = 75).

	Mean or N	SD or %
Age in years, mean (SD)	63.7 (range: 20–90)	12.3
Age group, n (%)		
< 65 years	495	42.4
≥65 years	672	57.6
Gender, n (%)		
Women	790	67.7
Men	369	31.6
Other	8	0.7
Educational level, n (%)		
Low	64	5.5
Intermediate	507	43.4
High	596	51.1
Marital status, n (%)		
Married or living together	775	66.4
Single or widowed	392	33.6
Source of recruitment, n (%)		
Via Dutch Brain Research Registry	1115	95.5
Via Dutch Memory Clinic Network	52	4.5
Visited a memory clinic, n (%)		
Yes	237	20.3
No or unsure	930	79.7
Self-reported dementia knowledge, n (%)		
Excellent, good or considerable	513	44.0
Limited or poor	654	56.0

**Figure 1.**
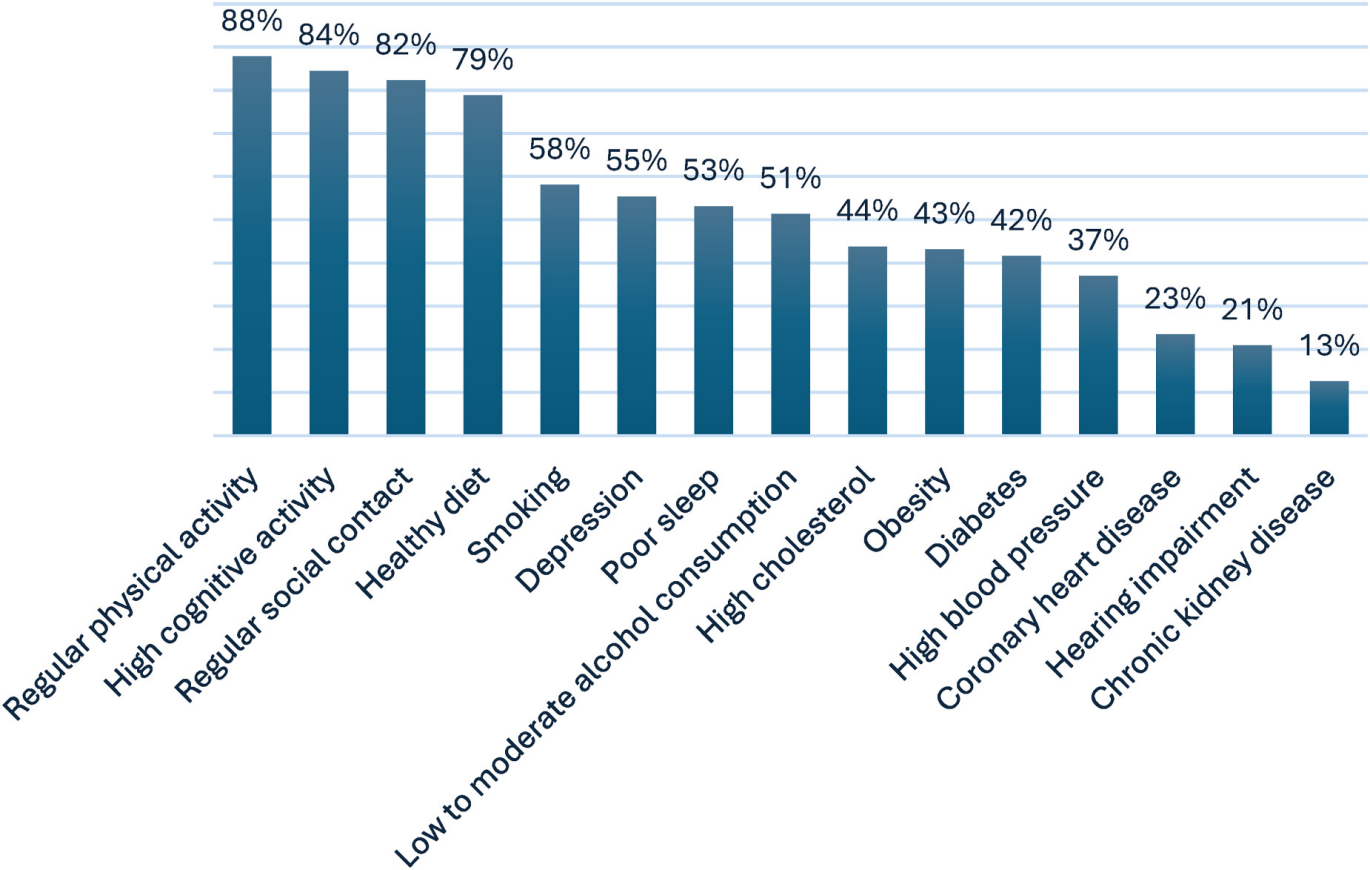
Correctly identified dementia risk and protective factors by surveyed participants with SCD (n = 1092) and MCI (n = 75).

*Dementia risk awareness.* Overall, 39.5% of SCD and MCI participants were unaware (i.e., agreed or doubted) that they could take action to lower their dementia risk. Full distribution of responses and stratified results by education level and prior memory clinic visit are provided in Supplemental Tables 1–3 (Supplemental Material 3). Participants aged <65 years (68.7%) were more aware than older participants (54.5%) (χ^2^(1) = 24.13, p < 0.001), and participants with high educational levels (65.7%) were more aware than those with intermediate (56.0%) or low (46.9%) educational levels (χ^2^(2) = 16.17, p < 0.001). Participants with SCD (61.4%) were more aware compared to those with MCI (48.0%) (χ^2^(1) = 5.24, p = 0.022). No differences were found based on gender, recruitment source, prior visit to a memory clinic and self-perceived dementia knowledge. Regular physical activity (87.8%), high cognitive activity (84.5%), and regular social contact (82.3%) were the dementia risk or protective factors most frequently identified by individuals with SCD and MCI. In contrast, coronary heart disease (23.5%), hearing impairment (20.9%), and chronic kidney disease (12.7%) were least frequently recognized (see Figure 1).

*Needs and preferences.* Most participants with SCD and MCI expressed interest in receiving information on improving brain health, answering “yes” (79.3%) or “maybe” (18.1%). Stratified results by education level and prior memory clinic visit are provided in Supplemental Tables 4 and 5 (Supplemental Material 3). Interest was higher among participants with higher (82.2%) and intermediate (77.6%) educational levels than those with lower education (64.3%; Fisher's exact, p = 0.013). Men (86.4%) were relatively more interested than women (76.1%) and gender-diverse (71.4%) participants (Fisher's exact, p < 0.001). No differences were found based on age, diagnosis, recruitment source, prior visit to a memory clinic and self-perceived dementia knowledge among individuals with SCD and MCI. In the multiple-choice question on preferred information sources, participants most often selected an open internet search (63.2%), the Alzheimer's Society website (43.4%) and consulting a general practitioner or practice nurse (36.7%).

A combination of formats (e.g., internet and printed materials) was most preferred (46.5%), followed by the internet (35.6%), other methods (10.3%), and printed brochures (7.6%). Stratified results by education level and prior memory clinic visit are provided in Supplemental Tables 6 and 7 (Supplemental Material 3). Older participants (≥65 years) more often preferred a combination (49.5%) than internet only (30.95%), while younger participants showed equal preference for both (42.4% and 42.0%, respectively; χ^2^(3) = 16.40, p = 0.001). Preference for printed materials was higher among lower (10.7%) and intermediate (9.6%) educated participants than among higher educated (5.6%; Fisher's exact, p = 0.019). Internet preference was greater among participants who had not or were unsure if they had visited a memory clinic compared to those who did (37.5% versus 27.9%; χ^2^(6) = 12.68, p = 0.005). No differences were found by gender, diagnosis, recruitment source, or self-perceived dementia knowledge. Most participants (81.2%) indicated they would use a free website or app on brain health; 16.7% responded “maybe”. Stratified results by education level and prior memory clinic visit are provided in Supplemental Tables 8 and 9 (Supplemental Material 3). Older participants (≥65 years) were more likely to respond “yes” (82.6%) than younger participants (79.3%), who more often said “maybe” (19.6% versus 14.5%) (χ^2^(2) = 9.30, p = 0.010). Men expressed greater interest (“yes”, 87.0%), than women (78.6%) and gender-diverse participants (71.4%; Fisher's exact, p = 0.006). No differences were found based on education, diagnosis, prior visit to a memory clinic, recruitment source, and self-perceived dementia knowledge. Most reported barriers to improving brain health were a lack of knowledge (51.2%) and motivation (23.2%). Lack of knowledge was more reported among participants who had not visited a memory clinic (50.8% versus 41.8%, χ^2^(1) = 6.10, p = 0.014) and those reporting limited or poor dementia knowledge (56.6% versus 39.2%, χ^2^(1) = 34.81, p < 0.001). Lack of motivation was more often reported by participants who had not visited a memory clinic (24.2% versus 14.8%, χ^2^(1) = 9.70, p = 0.002). No other differences were found based on education, diagnosis, prior visit to a memory clinic, and recruitment source.

### Survey of memory clinic professionals

Professionals not working at a memory clinic (n = 5) were excluded, resulting in 58 professionals for further study (see Table 2 for demographics). All proportions are reported based on the number of respondents per item; item-level missing data were modest (≤6.9%) and are therefore not reported separately.

**Table 2.** Demographics of survey memory clinic professionals (N = 58).

	Mean or N	SD or %
Mean age, years (SD)	41.8 (range: 24–68)	11.9
Gender, n (%)		
Women	49	84.5
Men	9	15.5
Other	0	-
Educational level, n (%)		
Low	0	-
Intermediate	5	8.6
High	53	91.4
Profession, n (%)		
Psychologist	15	25.9
Physician or medical specialist	30	51.7
Nurse or nurse practitioner	9	15.5
Other	4	6.9
Years working at a memory clinic, n (%)		
Less than 1 year	6	10.3
1 to 5 years	26	44.8
6 to 10 years	7	12.1
11 to 20 years	13	22.4
More than 20 years	6	10.3
Memory clinic location, n (%)		
General hospital	27	46.6
Academic hospital	25	43.1
Mental health care (GGZ) or a combination of work locations	6	10.3
Self-reported dementia knowledge, n (%)		
Excellent, good or considerable	50	86.2
Limited or poor	8	13.8

**Figure 2.**
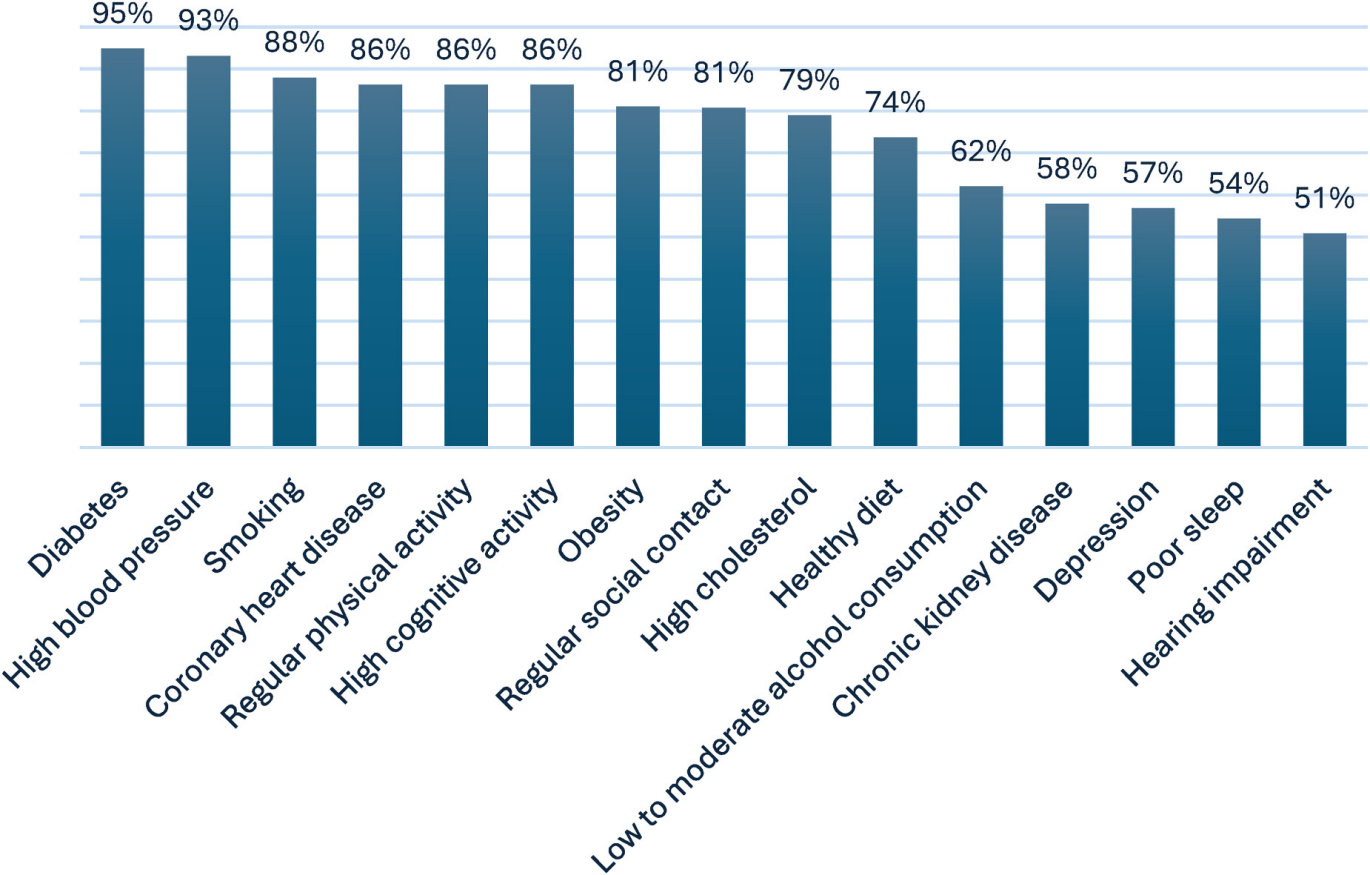
Correctly identified dementia risk and protective factors by surveyed memory clinic professionals (N = 58).

*Risk awareness of dementia risk reduction.* Overall, 10.3% of memory clinic professionals were unaware (i.e., agreed or doubted) that action could be taken to lower dementia risk. Full distribution of responses is provided in Supplemental Table 10 (Supplemental Material 3). No differences were found based on professionals’ age, educational level, gender, profession or workplace setting. The most frequently correctly identified dementia risk and protective factors were diabetes (94.9%), hypertension (93.2%), and smoking (88.1%). In contrast, depression (56.9%), poor sleep (54.4%), and hearing impairment (50.9%) were the least frequently recognized factors, albeit still by more than half of professionals (Figure 2).

*Needs and preferences.* Most memory clinic professionals reported that they always (32.7%), regularly (43.6%), or sometimes (18.2%) provide information on dementia risk reduction during consultations with patients with SCD and MCI, while some never do (5.5%). Professionals working in general hospitals (51.9%) were significantly more likely to ‘’always’’ provide such information than those in academic hospitals (17.4%) or other work environments (0%, Fisher's exact, p = 0.020). Workplace setting was not significantly associated with whether they provided information at all. In addition, no differences were found based on professionals’ age, education, gender, or profession. Those who provided information most frequently discussed physical activity (92.3%), cognitive activity (80.8%), alcohol consumption (75.0%), and social activity (73.0%). Diabetes (22.2%), high cholesterol (17.3%), coronary heart disease (11.5%) and chronic kidney disease (5.8%) were least frequently discussed.

All but one professional (98.0%) reported providing risk reduction information in a personalized way (76.5% “always”, 21.6% “sometimes”). This personalization was typically based on information gathered during patient anamnesis (74.0%) or diagnostic test results (12%), and 72.6% indicated using supporting materials (websites, apps, printed resources) regularly during risk reduction discussions. Professionals with an intermediate level of education (100%) were more likely to regularly use supporting materials than those with a higher level of education (70.2%, Fisher's exact, p = 0.040). Similarly, those working in general hospitals (80.8%) more frequently used such materials than their counterparts in academic hospitals (65%) or other work environments (60%, Fisher's exact, p = 0.026). No differences were found based on professionals’ age, gender, or profession. Furthermore, 87.0% of professionals expressed interest in using a website or app specifically focused on dementia risk reduction through lifestyle changes when counselling patients with SCD and MCI at the memory clinic. Additionally, 94.6% believed that their patients have (“yes”) or possibly have (“maybe”) a need for such digital tools to address dementia risk reduction. No differences were found based on professionals’ age, gender, education, profession or workplace setting.

### Interviews

We interviewed fourteen individuals (mean age: 65.3; SD: 13.2; 36% female) with SCD (n = 9) and MCI (n = 5) who had visited a memory clinic and nine memory clinic professionals (mean age: 44.2; SD: 11.9; 78% female) consisting of four nurses, three physicians and two psychologists. Thematic analysis identified three main themes: risk reduction information needs, its method of delivery and implementation of materials on this topic. Themes are presented below, accompanied by illustrative quotes.

*Information needs regarding dementia risk reduction.* All participants with SCD and MCI were, to a certain extent, interested in understanding how modifiable risk and protective factors relate to dementia risk. More specifically, they wanted to know whether making lifestyle changes could influence their individual prognosis. Memory clinic professionals acknowledged this need, stating that having easy access to evidence-based information on dementia risk reduction would be useful in practice as it is a common question by patients with SCD and MCI, adding that it would help them structure their conversations on dementia risk reduction and provide informed advice to patients. Both groups emphasized that information on this topic should be in-depth, up-to-date, and evidence-based. They further highlighted the importance of gaining insight into how specific risk and protective factors influence brain health (e.g., underlying mechanisms), from an official source, such as a university or patient organization, as this would enhance the perceived trustworthiness of the content. Alongside the information, practical and feasible tips were deemed essential to motivate individuals to apply knowledge of modifiable factors and to make sustainable brain-healthy lifestyle choices.

“*[I want to know] which factors are truly important and which ones I can influence myself. Like smoking, drinking, exercise. […] [Tips] should also just be feasible. […] You know, you can say, ‘You should never eat salt,’ but if you're at a birthday and someone has cooked for you, it's incredibly rude to say, ‘No, I don’t want that.’ People just won’t do that. So, I think that's really important – that it gives a realistic idea of what you can actually do.” – Participant with SCD, female, 24 years old*

Additionally, participants with SCD and MCI preferred getting concrete insights into the personal short-term benefits of lifestyle changes, as otherwise the concept of dementia risk reduction felt somewhat abstract. In that vein, it was mentioned that stressing its relevance to their current lives could support motivation to adopt brain-healthy behaviors.

“*What do I gain by doing [lifestyle changes]? Because people – me included – need to feel motivated to take action.” – Participant with MCI, female, 63 years old*

Participants with SCD and MCI also expressed a need for receiving more general information on managing daily challenges, such as forgetfulness, and informing those around them about their symptoms, alongside more specific information on the influence of modifiable dementia risk and protective factors.

*How risk reduction information should be delivered.* All participants with SCD and MCI responded positively to online information on dementia risk reduction, valuing its accessibility and flexibility for consultation. Many felt comfortable using the internet for information gathering but noted that offline materials are also necessary for those with lower digital skills. Memory clinic professionals were also open to online formats, recognizing that digital delivery aligns with current healthcare trends. They, too, expressed concerns about digital literacy, especially in their older patient population, stressing the need for offline materials to keep information on dementia risk reduction accessible to all.

“*In this digital age, we notice that some patients are simply unable to keep up and that they really appreciate being able to read things over at home [on paper]. […] So, I think it should be a mix [of online and offline information].” – Physician, female, 33 years old*

Both participants with SCD and MCI and memory clinic professionals preferred information to be presented in a layered way, starting with basic explanations and allowing users to access more in-depth content if desired. This approach was suggested to avoid information-overload. Participants also valued diverse presentation formats, such as visuals, videos, infographics, or gamified elements, to support comprehension and engagement, especially for those who found reading long texts more difficult due to memory problems.

“*I've noticed that I increasingly prefer videos. They also stick better. That has to do with my memory [problems]. Visually, everything still works really well. What I see, I can generally retain. But texts, especially long pieces of text, I tend to zone out.” – Participant with MCI, male, 66 years old*

Both groups of participants emphasized that the language must be simple and easy to understand yet not childish or patronizing. The tone should remain friendly, positive and nuanced, recognizing the realities and emotional weight of living with memory problems without becoming overly heavy. Professionals particularly emphasized the importance of avoiding any victim blaming and not to unintentionally evoke feelings of guilt, as such messaging was not only considered inaccurate but also potentially harmful and demotivating.

“*I sometimes find it difficult…* *that people might feel like, ‘Oh, this is my fault, I caused this, and now I have to do everything I can to fix it.’ Whereas it's also important to take that burden of guilt away. So, the information [on dementia risk reduction] should be presented with a positive approach. […] So that people feel a bit empowered” – Physician, female, 36 years old*

Accordingly, terminology preferences reflected this concern, with both participant groups generally preferring “brain health” over “dementia risk” due to its less stigmatizing and non-blaming connotation. Both groups further emphasized the value of personalized information, as it helped users focus on their personalized risk profile and could increase motivation to act. Many participants, especially professionals, also saw individual coaching as a helpful, optional addition, noting that information alone often falls short in supporting behavior change. It was important that coaching involved a real person and not an AI tool. For all functions included in a risk management tool, participants with SCD and MCI stressed the need for autonomy and flexibility, noting that imposed elements, such as mandatory personalization or coaching, could discourage engagement with the tool.

*Implementation.* Both participants with SCD and MCI and memory clinic professionals expressed enthusiasm for the potential use of dementia risk reduction materials in a memory clinic setting. They believed these materials could enhance care for individuals experiencing memory problems. One participant with SCD expressed strong interest when introduced to the idea of online materials offering information about dementia risk reduction in a memory clinic setting, responding, “*Absolutely, yes,”* and explaining:

“*Because I want to do as much as I can to age healthily.” – Participant with MCI, male, 70 years old*

Participants agreed that all healthcare professionals involved in memory clinic care could offer these materials. However, professionals noted that the timing of introducing the topic of risk reduction was crucial and would depend on specific workflows at each clinic. Sometimes, patients are seen only once, typically during a results consultation, which may be emotionally charged due to potential disclosure of a diagnosis and therefore not ideal to introduce lifestyle-related topics. Limited consultation time further reduced the opportunity to introduce such materials effectively. When a healthcare professional has more frequent contact with the patient, they were perceived as better positioned to discuss the materials.

*Lack of time [is a barrier], because there are often other issues that require more immediate attention. […] Ideally, you would have a separate session just about lifestyle factors — really taking the time to explore how someone manages these things. But sometimes other matters take priority in the moment over lifestyle. – Psychologist, female, 45 years old*

Professionals also raised concerns about how to ensure consistent implementation across memory clinics. Additionally, professionals expressed concern about potential costs for using materials, external coaching components, and offering follow-up appointments to support behavior change themselves. Although procedures, roles, available time and funds differ across clinics, potentially complicating integration, many professionals believed that nationwide, uniform implementation would promote uptake, clarity, and consistent use of the materials in practice. Professionals recommended developing the materials and their dissemination with established organizations, like national Alzheimer societies, to ensure recognizability and ease of integration. Fragmentation across multiple platforms or sources was seen as confusing and counterproductive.

“*The most important thing, I think, is that you need to avoid ending up with six different websites and six different apps for this target group. […] That makes things very confusing. And when it becomes confusing, we [healthcare professionals] often end up saying: never mind. So, if you're going to develop something like this, I would recommend doing it in collaboration with existing organizations. […] That way, we know, for example: we’ve always worked with materials from Alzheimer Netherlands. But now we have specific Alzheimer Netherlands materials for MCI. […] I think that's really important. – Nurse, female, 40 years old*

## Discussion

This explanatory mixed-methods study aimed to understand risk awareness and identify the needs and preferences for receiving information on dementia risk reduction among individuals with SCD and MCI in memory clinic and community settings, as well as memory clinic professionals. Findings show a need for developing evidence-based materials for clinical practice, as shown by evident knowledge gaps, current lack of trusted resources, and personal interest in felt relevance of the topic. Several recommendations for creating such material, in print and online, were provided by both groups, with the aim of maximizing acceptability, fit to existing needs and wishes, and scalability.

Our survey findings indicated that a large proportion of individuals with SCD and MCI, predominantly community-based research volunteers with approximately one in five having attended a memory clinic, were unaware that dementia risk can be reduced, particularly among older adults and those with lower educational attainment. Notably, this low level of awareness was observed despite the relatively high educational attainment of our sample, suggesting that awareness may be even lower in typical memory clinic populations. This is consistent with earlier studies reporting limited awareness of dementia risk reduction among middle-aged and older adults in the general population.^8,9,11–13^^,30^ In contrast, most memory clinic professionals demonstrated good awareness of modifiable dementia risk and protective factors, which is supported by previous studies among health care professionals.^31,32^ While individuals with SCD and MCI often linked lifestyle factors such as physical activity, cognitive engagement, social contact, and healthy diet to dementia risk, they less frequently recognized cardiovascular conditions, like hypertension and diabetes, as relevant risk factors for dementia. This mirrors prior findings, indicating that the contribution of cardiovascular health to dementia risk is generally underestimated.^8,10,30^ Professionals consistently recognized these factors more often, aligning with a previous study among Dutch general practitioners and practice nurses who correctly identified cardiovascular factors as dementia risk factors.^32^ However, despite this awareness, our survey showed that professionals more often discussed lifestyle factors during conversations, suggesting that the cardiovascular aspect of dementia risk may nevertheless be underemphasized in clinical consultations. Our findings highlight the need to enhance communication on dementia risk reduction for those with SCD and MCI in memory clinics and broader community settings, and more attention should be given to the role of cardiovascular risk factors.

Our survey showed that almost all individuals with SCD and MCI were interested in receiving information on how to improve their brain health. However, interest was lower among individuals with lower educational attainment, which aligns with health literacy research showing that lower educational levels are linked to greater challenges in accessing, understanding, appraising, and applying health information, and may help explain the reduced interest observed in this group.^33,34^ A majority, but particularly older individuals and men, reported that they would consider using a free digital tool aimed at reducing dementia risk. This highlights a clear demand for preventive guidance in this population. A previous study found similar results and reported that 98% of participants with SCD expected to use “an online program providing advice on lifestyle to maintain brain health”.^26^ The high level of interest in digital tools in our study should be interpreted in light of the study sample, which largely consisted of digitally engaged research volunteers with higher educational attainment. Consequently, reported interest in digital tools may be higher than would be expected in more typical memory clinic populations, particularly among individuals with lower education, digital literacy, or access to resources. Memory clinic professionals also showed strong interest in materials on dementia risk reduction, noting its potential to help structure conversations around this topic and provide patients with reliable, consistent information. They confirmed that digital tools might be helpful in supporting their patients in lifestyle-based dementia prevention. eHealth interventions, including online tools, have increasingly been recognized as promising methods for raising awareness and promoting behavior change,^24,35,36^ and their potential contribution to dementia risk reduction is gaining attention.^25,37^ Moreover, recent studies show that the general population actively seeks information on dementia risk reduction through online resources, and that such tools could improve knowledge of brain health.^12,13,24^ Our survey revealed that both groups were enthusiastic about digital formats, but in-depth interviews further indicated that offline materials are deemed an essential addition for those with limited digital skills. Most SCD and MCI participants, particularly older individuals, preferred receiving information on dementia risk reduction through a combination of online and offline formats. Prior studies underline the necessity for offline options in health education, indicating that older adults may encounter digital obstacles and favor a combination of offline and online formats because of factors like access, trust, and digital literacy.^38–41^

Participants with SCD and MCI frequently flagged insufficient knowledge of dementia risk reduction and a lack of motivation in our survey as the most important barriers to enhancing their brain health, consistent with barriers for adopting healthy lifestyle changes found in other patient populations.^42^ They further explained in our interviews that for an online dementia risk reduction tool to be acceptable and useful for gathering knowledge, it must meet certain criteria: the sender must be credible, information should be up-to-date and personalized, the tool should allow autonomy (e.g., no mandatory pathways or access restrictions), and the interface must be user-friendly and inviting. This is supported by prior research including similar participants on an online lifestyle program for individuals with SCD, identifying trustworthiness, user-friendliness, personalization, and flexibility as key facilitators for use.^26^ Our interviews also revealed that many participants with SCD and MCI wanted additional, general guidance on coping with memory challenges, and both participant groups advocated for coaching to implement lifestyle advice into daily routines. Incorporating elements of coaching in eHealth interventions has previously proved to be more effective than purely digital approaches when it comes to sustained lifestyle behavior changes. For example, digital interventions with coaching reduced diabetes risk in midlife and older adults by 37%, compared to 12% in stand-alone digital interventions,^43^ and incorporating minimal levels of human contact has been linked to improved adherence to digital self-help interventions among family caregivers of people with dementia.^44^

Our participants with SCD and MCI and memory clinic professionals emphasized avoiding guilt-based messages that suggest personal responsibility for memory problems, as such victim blaming could be harmful and demotivating. Indeed, prior research on ethical aspects of dementia prevention cautioned that emphasizing personal responsibility can foster self-blame and stigma, which may decrease motivation for lifestyle changes.^45^ In line with this, participants preferred terminology such as “brain health” over “dementia risk”, as it was perceived as less stigmatizing and more supportive of a non-blaming framing. Lastly, memory clinic professionals saw time constraints as a potential barrier to integrating such tools into their routine care and elaborated that implementation would be more feasible if materials were rolled out consistently nationwide and embedded within established healthcare structures. Previous research among healthcare professionals in cardiac and primary care has identified similar factors when working with eHealth for prevention purposes, suggesting that these factors are not unique to our context.^46,47^

Based on the integrated survey and interview findings, key recommendations for dementia risk reduction communication and tool development are presented in Box 1.

Box 1. Key implications for dementia risk reduction communication

1. Dementia risk reduction should be routinely communicated in care settings for individuals with SCD and MCI with clear, up-to-date, evidence-based information that includes cardiovascular risk factors alongside lifestyle-related behaviors.
2. Communication should adopt supportive, non-blaming language and framing (e.g., by using “brain health” over “dementia risk”).
3. Digital delivery of information is appreciated, but should be embedded within hybrid formats that are complemented by offline materials to ensure accessibility across age groups, educational levels, and digital literacy.
4. Digital tools should be designed to support autonomy and feasibility, prioritizing trustworthiness, usability and integration within existing healthcare structures.

### Strengths and limitations

Our study has some notable strengths. First, the mixed-methods design enabled a comprehensive understanding of risk awareness, needs, and preferences among individuals with SCD and MCI and memory clinic professionals, combining quantitative insights from a large sample with qualitative exploration of barriers and facilitators. Second, the inclusion of a substantial number of participants strengthened the quantitative survey findings. Investigator triangulation, independent coding by two researchers, iterative refinement of the interview process, and data saturation enhanced the trustworthiness and robustness of our qualitative interview findings.^48^ Third, we included individuals with SCD and MCI from all parts of the Netherlands, whether they sought care from memory clinics or not, thus enhancing the diversity and representativeness of our sample. Fourth, memory clinic professionals from academic hospitals, general hospitals, and mental health care (GGZ) with varied professional backgrounds and levels of work experience were included, further supporting the generalizability and applicability of our findings across different clinical settings. However, some limitations must be considered when interpreting our findings. All questionnaires were distributed online, and we recruited most participants with SCD and MCI through the online Dutch Brain Research Registry. This may have introduced selection bias by mainly including those with higher digital literacy and internet access, limiting our findings’ representativeness and generalizability. The underrepresentation of individuals with SCD and MCI with lower educational levels further restricts generalizability, possibly neglecting the unique barriers and needs of this group and exacerbating health inequalities. However, we were still able to study differences by educational background in this sample. Although gender-diverse participants were included in gender comparison analyses for inclusivity, the small subgroup size limits the precision and interpretability of the estimates for this group. Future studies should recruit larger, more diverse samples of individuals with SCD and MCI (specifically those with limited digital skills and lower education levels) to improve generalizability and promote inclusive online dementia risk reduction initiatives. This may be facilitated through targeted outreach, the inclusion of paper-based pathways alongside digital approaches and prioritizing co-design with individuals with lower health literacy.^49^

### Conclusion

This study shows a need by individuals with SCD and MCI in memory clinic and community settings and memory clinic professionals for developing evidence-based material to improve communication about dementia risk reduction, particularly for addressing gaps in risk awareness among those with SCD and MCI and by strengthening attention to the contribution of cardiovascular risk factors to dementia risk. Both individuals with SCD and MCI and memory clinic professionals expressed interest in digital tools to support dementia risk reduction. Such tools should be easily accessible, user-friendly, and presented in a non-judgmental tone. To maximize reach and impact, tools should offer personalized information, coaching, be accompanied by offline materials, and be embedded within established healthcare structures. By outlining specific needs and preferences, this study offers guidance for the development and integration of eHealth interventions for dementia risk reduction into routine memory clinic practice.

## Supplemental Material

sj-docx-1-alz-10.1177_13872877261440958 - Supplemental material for Towards specialized dementia risk reduction services for those with first cognitive symptoms: A mixed-method study into risk awareness, needs, and preferences among individuals with subjective cognitive decline and mild cognitive impairment from memory clinic and community settings and memory clinic professionals Supplemental material, sj-docx-1-alz-10.1177_13872877261440958 for Towards specialized dementia risk reduction services for those with first cognitive symptoms: A mixed-method study into risk awareness, needs, and preferences among individuals with subjective cognitive decline and mild cognitive impairment from memory clinic and community settings and memory clinic professionals by Lotte S. Truin, Irene S. Heger, Sophie C. P. M. Wimmers, Kay Deckers, Marjolein E. de Vugt and Sebastian Köhler in Journal of Alzheimer's Disease

sj-docx-2-alz-10.1177_13872877261440958 - Supplemental material for Towards specialized dementia risk reduction services for those with first cognitive symptoms: A mixed-method study into risk awareness, needs, and preferences among individuals with subjective cognitive decline and mild cognitive impairment from memory clinic and community settings and memory clinic professionals Supplemental material, sj-docx-2-alz-10.1177_13872877261440958 for Towards specialized dementia risk reduction services for those with first cognitive symptoms: A mixed-method study into risk awareness, needs, and preferences among individuals with subjective cognitive decline and mild cognitive impairment from memory clinic and community settings and memory clinic professionals by Lotte S. Truin, Irene S. Heger, Sophie C. P. M. Wimmers, Kay Deckers, Marjolein E. de Vugt and Sebastian Köhler in Journal of Alzheimer's Disease

sj-docx-3-alz-10.1177_13872877261440958 - Supplemental material for Towards specialized dementia risk reduction services for those with first cognitive symptoms: A mixed-method study into risk awareness, needs, and preferences among individuals with subjective cognitive decline and mild cognitive impairment from memory clinic and community settings and memory clinic professionals Supplemental material, sj-docx-3-alz-10.1177_13872877261440958 for Towards specialized dementia risk reduction services for those with first cognitive symptoms: A mixed-method study into risk awareness, needs, and preferences among individuals with subjective cognitive decline and mild cognitive impairment from memory clinic and community settings and memory clinic professionals by Lotte S. Truin, Irene S. Heger, Sophie C. P. M. Wimmers, Kay Deckers, Marjolein E. de Vugt and Sebastian Köhler in Journal of Alzheimer's Disease
