# Supplementary material for: Towards specialized dementia risk reduction services for those with first cognitive symptoms: A mixed-method study into risk awareness, needs, and preferences among individuals with subjective cognitive decline and mild cognitive impairment from memory clinic and community settings and memory clinic professionals
Source: J Alzheimers Dis. 2026 Apr 17;111(3):1095–107. doi: 10.1177/13872877261440958 (PMC13219763; doi:10.1177/13872877261440958)
Supplement: sj-docx-1-alz-10.1177_13872877261440958 - Supplemental material for Towards specialized dementia risk reduction services for those with first cognitive symptoms: A mixed-method study into risk awareness, needs, and preferences among individuals with subjective cognitive decline and mild cognitive im [file sj-docx-1-alz-10.1177_13872877261440958.docx]

**Supplemental Material 1 – Online surveys for individuals with SCD and MCI and memory clinic professionals**

*Survey for individuals with SCD and MCI*

**INFORMED CONSENT**

Dear reader,

Thank you for your interest in our research. We are inviting you to participate in a scientific study. Participation is voluntary. To participate, we need your consent.

Before you decide whether or not to take part in the study, we will explain what the research involves.

Please read this information carefully and feel free to ask the researcher for further explanation if needed.

**Background of the research**

Maastricht University and the Alzheimer Center Limburg are developing a tool that provides information on what you can do yourself to keep your brain healthy and reduce the risk of dementia.

This tool is intended for people who have **cognitive complaints** and/or a diagnosis of **mild cognitive impairment**, but not dementia. The tool does not yet exist and still needs to be developed. To do that, we need your help.

We are interested in what you already know about keeping the brain healthy and reducing the risk of dementia. We would also like to hear what motivates or discourages you from using such a tool.

**Nature of the research**

This research consists of a questionnaire. You will complete the questionnaire online. This will take about 10 to 15 minutes. You may stop participating in the research at any time. You do not need to provide a reason for this.

**Confidentiality of your data**

In this study, we will collect some personal data. For example, your age and level of education. Collecting, using, and storing your data is necessary so that we can properly answer the research questions and publish the results. In reports and publications about the research, your data cannot be traced back to you.

**Data storage**

Your data will be securely stored within the research organization for a maximum of 15 years after collection. This is standard in scientific research. Your data may still be relevant for other scientific research after this study. For this, your data does not need to be stored longer than the usual 15 years.

**Withdrawing consent**

You can withdraw your consent for the use of your personal data at any time. This applies to this research, as well as to storing and using your data for future research. The research data collected up to the point at which you withdraw your consent will still be used in this study.

**More information about your data rights**

For general information about your rights when your personal data is used, you can visit the website of the Dutch Data Protection Authority (<https://www.autoriteitpersoonsgegevens.nl>). If you have questions about your rights, you can contact the lead researcher responsible for processing your personal data.

**What are the benefits of participating?**

Your participation in this study contributes to scientific research aimed at keeping the brain healthy. The information you give us helps us inform people with memory complaints about this topic. Healthcare professionals can also use this tool in the future in daily practice.

**What are the disadvantages of participating?**

The only disadvantage of this research is that you spend time filling out the questionnaire. There are no other disadvantages to this study.

**Do you have questions?**

If you have questions you can contact Lotte Truin by phone (+31 433 88 10 48) or email (l.truin@maastrichtuniversity.nl).

**Research "Health and Dementia"**

I have been informed about the study.
I have read the written information provided before this questionnaire study.
I have had the opportunity to ask questions about the study.
I have had time to consider my participation, which is entirely voluntary.
I understand that I have the right to withdraw my consent at any time and stop participating in the study without giving a reason.

- I consent to participate in the study
- I do not consent to participate in the study

**DEMOGRAPHIC INFORMATION**

How old are you?

What is your gender?

Male

Female

Other, namely:

Prefer not to say

In which province do you live?

Groningen

Friesland

Drenthe

Overijssel

Flevoland

Gelderland

Utrecht

North-Holland

South-Holland

Zeeland

Brabant

Limburg

What is the highest level of education you have completed (with a diploma or certificate)?

No education (did not complete primary education)
Primary education (elementary school, special elementary education)
Lower or preparatory vocational education (e.g., trade school, domestic science school, lts, leao, lhno, vmbo-b/k)
Lower general secondary education (e.g., (m)ulo, mavo, vmbo-g/t, short mbo, mbo-1)
Intermediate vocational and dual education (e.g., vocational training for baker or hairdresser, long mbo, mts, uts, meao, bol, bbl, inas, mbo-2, mbo-3, mbo-4)
Higher general and pre-university education (e.g., hbs, mms, havo, vwo, atheneum, gymnasium)
Higher professional education (e.g., teacher training college, hbo, hts, heao, hbo nursing, university bachelor's)
University education (doctoral or master’s degree, postdoctoral, hbo master's)

What is your marital status?

Married/registered partnership
Cohabiting
Unmarried, never married
Divorced/separated
Widowed

How would you rate your health?

Excellent
Good
Fair
Moderate
Poor

According to our records, you are known at the memory clinic.

**How many times have you visited the memory clinic so far?**

I have never been to the memory clinic
I had one appointment at the memory clinic
I had two or more appointments at the memory clinic

Why did you go to the memory clinic?

I have complaints about my thinking abilities (such as memory problems, concentration issues, or language difficulties), but I don't know (yet) what is going on.
I have complaints about my thinking abilities (such as memory problems, concentration issues, or language difficulties), but **no cognitive disorder** was diagnosed.
A mild cognitive disorder has been diagnosed (also known as **mild cognitive impairment**).
**Dementia** has been diagnosed.
I don’t know why I went to the memory clinic.
Other, namely:

**KNOWLEDGE OF DEMENTIA**

Dementia consists of a collection of symptoms, most notably memory loss.

In addition, sudden mood changes, forgetting who people are, and not being able to find words are common. Alzheimer’s disease is one form of dementia.

**How would you rate your own knowledge of dementia?**

Excellent
Good
Fair
Limited
None

**Outside of your paid work, what do you currently do or what have you done in the past for someone with dementia?**

Check all answers that apply.

Caring for someone (other than myself) who lives/lived with me

Regularly caring for someone who does/did **not** live with me

Being responsible for making appointments for someone who needs care (for example: arranging a visit somewhere or finding a place in a care home)

Occasionally helping someone

Encouraging someone with symptoms of dementia to seek professional help

Occasionally visiting someone

Other, namely:

None of the above

The following questions are about ways to reduce the risk of dementia. This specifically concerns reducing the risk of dementia in people who already have **cognitive complaints** but have **not** been diagnosed with dementia.

To what extent do you agree or disagree with the following statements?

**'There is nothing you can do to lower your risk of dementia.'**

Strongly agree
Agree
Neither agree nor disagree
Disagree
Strongly disagree

**'High blood pressure increases the risk of developing dementia.'**

Strongly agree
Agree
Neither agree nor disagree
Disagree
Strongly disagree

**'Smoking increases the risk of developing dementia.'**

Strongly agree
Agree
Neither agree nor disagree
Disagree
Strongly disagree

**'Not drinking alcohol or drinking only a small amount lowers the risk of dementia.'**

Strongly agree
Agree
Neither agree nor disagree
Disagree
Strongly disagree

**'Regular physical activity lowers the risk of dementia.'**

Strongly agree
Agree
Neither agree nor disagree
Disagree
Strongly disagree

The following questions are about ways to reduce the risk of dementia. This specifically concerns reducing the risk of dementia in people who already have **cognitive complaints** but have **not** been diagnosed with dementia.

To what extent do you agree or disagree with the following statements?

**'Depression increases the risk of dementia.'**

Strongly agree
Agree
Neither agree nor disagree
Disagree
Strongly disagree

**'Diabetes increases the risk of dementia.'**

Strongly agree
Agree
Neither agree nor disagree
Disagree
Strongly disagree

**'Being overweight increases the risk of dementia.'**

Strongly agree
Agree
Neither agree nor disagree
Disagree
Strongly disagree

**'Being mentally active lowers the risk of dementia.'**

Strongly agree
Agree
Neither agree nor disagree
Disagree
Strongly disagree

**'Heart disease increases the risk of dementia.'**

Strongly agree
Agree
Neither agree nor disagree
Disagree
Strongly disagree

The following questions are about ways to reduce the risk of dementia. This specifically concerns reducing the risk of dementia in people who already have **cognitive complaints** but have **not** been diagnosed with dementia.

To what extent do you agree or disagree with the following statements?

**'Kidney disease increases the risk of dementia.'**

Strongly agree
Agree
Neither agree nor disagree
Disagree
Strongly disagree

**'A high cholesterol level increases the risk of dementia.'**

Strongly agree
Agree
Neither agree nor disagree
Disagree
Strongly disagree

**'Healthy diet lowers the risk of dementia.'**

Strongly agree
Agree
Neither agree nor disagree
Disagree
Strongly disagree

**'Being socially active lowers the risk of dementia.'**

Strongly agree
Agree
Neither agree nor disagree
Disagree
Strongly disagree

**'Poor sleep increases the risk of dementia.'**

Strongly agree
Agree
Neither agree nor disagree
Disagree
Strongly disagree

**'Hearing damage/hearing loss increases the risk of dementia.'**

Strongly agree
Agree
Neither agree nor disagree
Disagree
Strongly disagree

Below are a number of factors that may play a role in the development of dementia.

**According to you, which of the following are the three most important factors in the development of dementia?**

Being mentally inactive
Unhealthy diet
Being socially inactive
Obesity (overweight)
Diabetes
High cholesterol
Excessive alcohol consumption
Kidney disease
Hearing damage/hearing loss
Little or no physical activity
Depression
High blood pressure
Smoking
Poor sleep
Heart disease

**NEEDS**

Would you be interested in information about how to improve your brain health?

Yes

Maybe

No

Suppose you would like to know more about your own brain health.

**How would you prefer to find this information?**

*Select all answers that apply.*

Via the internet (using a search engine)
Via my doctor or psychologist at the memory clinic
Via my general practitioner or practice nurse
Via the Alzheimer Netherlands website
Via the library
Other, namely:

Suppose you would like to know more about your own brain health.

**What would you prefer?**

A printed leaflet or brochure
Information on the internet (website, mobile app, video)
A combination of the above options
Other, namely:

Suppose you are considering doing something about your own brain health.

**What would prevent you from taking action to improve your brain health?**

*Select all answers that apply.*

Lack of knowledge about it
Lack of time
Financial problems
Lack of motivation
Hard to organize
Health problems
Other, namely:

Suppose a website or app (mobile application) was developed that gives you free information about your brain health and how to improve it.

**Would you want to use it?**

Yes

Maybe

No, because:

Maastricht University and RadboudUMC are conducting research to develop a tool with information about lifestyle and brain health for people who visit the memory clinic. We are very interested in your ideas about developing such a tool.

**May we contact you without obligation to participate in this study?**

Yes, you may contact me at the following email address:
No

**VVV**

As a thank-you for completing this questionnaire, we will raffle VVV gift cards among participants.

Would you like to be eligible for one of the VVV gift cards? Then please leave your email address here. Winners will be notified by email.

**END + CONTACT**

**Click the right arrow below to submit your answers.**

Do you have any questions or comments following this study? Then please leave them below.

If you have any questions, you can also contact Lotte Truin by phone (+31 433 88 10 48) or email ([l.truin@maastrichtuniversity.nl](mailto:l.truin@maastrichtuniversity.nl)). You may have complaints about the research. You can discuss these with one of the researchers involved.

**Contact details of the researchers**

Lotte Truin, MSc (executing researcher)

Phone number: +31 433 88 10 48

Email: l.truin@maastrichtuniversity.nl

Dr. Irene Heger (researcher)

Email: irene.heger@maastrichtuniversity.nl

Dr. Sebastian Köhler (principal investigator)

Email: s.koehler@maastrichtuniversity.nl

Prof. Dr. Marjolein de Vugt (principal investigator)

Email: m.devugt@maastrichtuniversity.nl

*Survey for memory clinic professionals*

**INFORMED CONSENT**

Dear reader,

Thank you for your interest in our research. We are inviting you to participate in a scientific study. Participation is voluntary. To participate, we need your consent.

Before you decide whether or not to take part in the study, we will explain what the research involves. Please read this information carefully and feel free to ask the researcher for further explanation if needed.

**Background of the research**

Maastricht University and the Alzheimer Center Limburg are developing a tool that provides information on what you can do yourself to keep your brain healthy and reduce the risk of dementia.

This tool is intended for people who have **cognitive complaints** and/or a diagnosis of **mild cognitive impairment**, but not dementia. The tool does not yet exist and still needs to be developed. To do that, we need your help.

We are interested in what you, as a healthcare professional, already know about how someone can maintain a healthy brain and reduce the risk of dementia. We would also like to hear what motivates you or discourages you from using such a tool.

**Nature of the research**

This research consists of a questionnaire. You will complete the questionnaire online. This will take about 10 to 15 minutes. You may stop participating in the research at any time. You do not need to provide a reason for this.

**Confidentiality of your data**

In this study, we will collect some personal data. For example, your age and level of education. Collecting, using, and storing your data is necessary so that we can properly answer the research questions and publish the results. In reports and publications about the research, your data cannot be traced back to you.

**Data storage**

Your data will be securely stored within the research organization for a maximum of 15 years after collection. This is standard in scientific research. Your data may still be relevant for other scientific research after this study. For this, your data does not need to be stored longer than the usual 15 years.

**Withdrawing consent**

You can withdraw your consent for the use of your personal data at any time. This applies to this research, as well as to storing and using your data for future research. The research data collected up to the point at which you withdraw your consent will still be used in this study.

**More information about your data rights**

For general information about your rights when your personal data is used, you can visit the website of the Dutch Data Protection Authority (<https://www.autoriteitpersoonsgegevens.nl>). If you have questions about your rights, you can contact the lead researcher responsible for processing your personal data.

**What are the benefits of participating?**

Your participation in this study contributes to scientific research aimed at keeping the brain healthy. The information you give us helps us inform people with memory complaints about this topic. Healthcare professionals can also use this tool in the future in daily practice.

**What are the disadvantages of participating?**

The only disadvantage of this research is that you spend time filling out the questionnaire. There are no other disadvantages to this study.

**Do you have questions?**

If you have questions you can contact Lotte Truin by phone (+31 433 88 10 48) or email (l.truin@maastrichtuniversity.nl).

**Research "Health and Dementia"**

I have been informed about the study.
I have read the written information provided before this questionnaire study.
I have had the opportunity to ask questions about the study.
I have had time to consider my participation, which is entirely voluntary.
I understand that I have the right to withdraw my consent at any time and stop participating in the study without giving a reason.

- I consent to participate in the study
- I do not consent to participate in the study

**DEMOGRAPHIC INFORMATION**

How old are you?

What is your gender?

Male

Female

Other, namely:

Prefer not to say

In which province do you live?

Groningen

Friesland

Drenthe

Overijssel

Flevoland

Gelderland

Utrecht

North-Holland

South-Holland

Zeeland

Brabant

Limburg

What is the highest level of education you have completed (with a diploma or certificate)?

No education (did not complete primary education)
Primary education (elementary school, special elementary education)
Lower or preparatory vocational education (e.g., trade school, domestic science school, lts, leao, lhno, vmbo-b/k)
Lower general secondary education (e.g., (m)ulo, mavo, vmbo-g/t, short mbo, mbo-1)
Intermediate vocational and dual education (e.g., vocational training for baker or hairdresser, long mbo, mts, uts, meao, bol, bbl, inas, mbo-2, mbo-3, mbo-4)
Higher general and pre-university education (e.g., hbs, mms, havo, vwo, atheneum, gymnasium)
Higher professional education (e.g., teacher training college, hbo, hts, heao, hbo nursing, university bachelor's)
University education (doctoral or master’s degree, postdoctoral, hbo master's)

What is your position?

Psychologist

Physician/Medical specialist

Nurse/Nurse specialist

Other, namely:

How many years of total work experience do you have in this role?
Less than 1 year
1 to 5 years
6 to 10 years
11 to 20 years
More than 20 years

How long have you been working at the memory clinic?
Less than 1 year
1 to 5 years
6 to 10 years
11 to 20 years
More than 20 years
I do not work at the memory clinic. I work here:

Are you working at the memory clinic in a general (regional) hospital or in a university hospital?
General hospital
University hospital
Other, namely:

**KNOWLEDGE OF DEMENTIA**

Dementia consists of a collection of symptoms, most notably memory loss.

In addition, sudden mood changes, forgetting who people are, and not being able to find words are common. Alzheimer’s disease is one form of dementia.

**How would you rate your own knowledge of dementia?**

Excellent
Good
Fair
Limited
None

**Outside of your paid work, what do you currently do or what have you done in the past for someone with dementia?**

Check all answers that apply.

Caring for someone (other than myself) who lives/lived with me

Regularly caring for someone who does/did **not** live with me

Being responsible for making appointments for someone who needs care (for example: arranging a visit somewhere or finding a place in a care home)

Occasionally helping someone

Encouraging someone with symptoms of dementia to seek professional help

Occasionally visiting someone

Other, namely:

None of the above

The following questions are about ways to reduce the risk of dementia. This specifically concerns reducing the risk of dementia in people who already have **cognitive complaints** but have **not** been diagnosed with dementia.

To what extent do you agree or disagree with the following statements?

**'There is nothing you can do to lower your risk of dementia.'**

Strongly agree
Agree
Neither agree nor disagree
Disagree
Strongly disagree

**'High blood pressure increases the risk of developing dementia.'**

Strongly agree
Agree
Neither agree nor disagree
Disagree
Strongly disagree

**'Smoking increases the risk of developing dementia.'**

Strongly agree
Agree
Neither agree nor disagree
Disagree
Strongly disagree

**'Not drinking alcohol or drinking only a small amount lowers the risk of dementia.'**

Strongly agree
Agree
Neither agree nor disagree
Disagree
Strongly disagree

**'Regular physical activity lowers the risk of dementia.'**

Strongly agree
Agree
Neither agree nor disagree
Disagree
Strongly disagree

The following questions are about ways to reduce the risk of dementia. This specifically concerns reducing the risk of dementia in people who already have **cognitive complaints** but have **not** been diagnosed with dementia.

To what extent do you agree or disagree with the following statements?

**'Depression increases the risk of dementia.'**

Strongly agree
Agree
Neither agree nor disagree
Disagree
Strongly disagree

**'Diabetes increases the risk of dementia.'**

Strongly agree
Agree
Neither agree nor disagree
Disagree
Strongly disagree

**'Being overweight increases the risk of dementia.'**

Strongly agree
Agree
Neither agree nor disagree
Disagree
Strongly disagree

**'Being mentally active lowers the risk of dementia.'**

Strongly agree
Agree
Neither agree nor disagree
Disagree
Strongly disagree

**'Heart disease increases the risk of dementia.'**

Strongly agree
Agree
Neither agree nor disagree
Disagree
Strongly disagree

The following questions are about ways to reduce the risk of dementia. This specifically concerns reducing the risk of dementia in people who already have **cognitive complaints** but have **not** been diagnosed with dementia.

To what extent do you agree or disagree with the following statements?

**'Kidney disease increases the risk of dementia.'**

Strongly agree
Agree
Neither agree nor disagree
Disagree
Strongly disagree

**'A high cholesterol level increases the risk of dementia.'**

Strongly agree
Agree
Neither agree nor disagree
Disagree
Strongly disagree

**'Healthy diet lowers the risk of dementia.'**

Strongly agree
Agree
Neither agree nor disagree
Disagree
Strongly disagree

**'Being socially active lowers the risk of dementia.'**

Strongly agree
Agree
Neither agree nor disagree
Disagree
Strongly disagree

**'Poor sleep increases the risk of dementia.'**

Strongly agree
Agree
Neither agree nor disagree
Disagree
Strongly disagree

**'Hearing damage/hearing loss increases the risk of dementia.'**

Strongly agree
Agree
Neither agree nor disagree
Disagree
Strongly disagree

Below are a number of factors that may play a role in the development of dementia.

**According to you, which of the following are the three most important factors in the development of dementia?**

Being mentally inactive
Unhealthy diet
Being socially inactive
Obesity (overweight)
Diabetes
High cholesterol
Excessive alcohol consumption
Kidney disease
Hearing damage/hearing loss
Little or no physical activity
Depression
High blood pressure
Smoking
Poor sleep
Heart disease

**COMMUNICATION ABOUT DEMENTIA RISK REDUCTION**

When it is determined that a patient has subjective or mild cognitive complaints, is there the possibility of a follow-up appointment with a healthcare professional from the memory clinic after the diagnosis conversation?

Yes, this is routinely offered
Yes, this is regularly offered
Yes, but this is rarely offered
No, this option is not available

Do you provide information about dementia risk reduction through lifestyle changes when patients visit you for subjective or mild cognitive complaints?

Yes, always
Yes, regularly
Yes, sometimes
No, never

Why not?

*Check all answers that apply.*

I do not know enough about dementia risk reduction through lifestyle changes
This topic is not relevant to patients
I think my patients are not interested in this information
It is difficult to talk about dementia risk reduction through lifestyle changes without making patients feel guilty
This topic is a lower priority
There is no time/opportunity to discuss dementia risk reduction through lifestyle changes
There is insufficient evidence on the effectiveness of dementia risk reduction through lifestyle changes
There are no guidelines I can use
Other, namely:

What do you give advice about?

*Check all answers that apply.*

High blood pressure
Smoking
Excessive alcohol use
Little or no physical activity
Depression
Diabetes
Obesity (overweight)
Being mentally active
Heart disease
Kidney disease
High cholesterol level
Unhealthy diet
Being socially inactive
Poor sleep
Hearing damage/hearing loss
Other, namely:

Do you personalize this information for your patients?

Yes

No

Sometimes

What do you base this personalization on?

Demographic information
Diagnostic test results
Information from medical history
Other, namely:

Do you use tools (such as printed materials, websites, apps, or a risk score) when providing information about dementia risk reduction to your patients?

Yes, with most patients
Yes, with some patients
Yes, but only if patients or their relatives request it
No, because:

**NEEDS**

Suppose a website or app (mobile application) was developed for patients at the memory clinic where personal information is provided about the room for lifestyle improvement by means of a risk score.

**In your experience at the memory clinic, is there a need for this among patients?**

Yes

Maybe

No

Suppose this website or app makes it easier to discuss lifestyle during a consultation. For example, by filling out a short questionnaire together and/or looking at a (visual) risk profile.

**Would you use this during a consultation? You may explain your answer.**

Yes

Maybe

No

Maastricht University and RadboudUMC are conducting research to develop an online tool with information about lifestyle and brain health for people who visit the memory clinic. We are very interested in your ideas about developing such a tool.

**May we contact you without obligation for a conversation about this?**

Yes, you may contact me at the following email address:
No

**END + CONTACT**

**Click the right arrow below to submit your answers.**

Do you have any questions or comments following this research? If so, please leave them below.

If you have any questions, you can also contact Lotte Truin by phone (+31 433 88 10 48) or email ([l.truin@maastrichtuniversity.nl](mailto:l.truin@maastrichtuniversity.nl)). You may have complaints about the research. You can discuss these with one of the researchers involved.

**Contact details of the researchers**

Lotte Truin, MSc (executing researcher)

Phone number: +31 433 88 10 48

Email: l.truin@maastrichtuniversity.nl

Dr. Irene Heger (researcher)

Email: irene.heger@maastrichtuniversity.nl

Dr. Sebastian Köhler (principal investigator)

Email: s.koehler@maastrichtuniversity.nl

Prof. Dr. Marjolein de Vugt (principal investigator)

Email: m.devugt@maastrichtuniversity.nl

Powered by Qualtrics
