# Supplementary material for: Towards specialized dementia risk reduction services for those with first cognitive symptoms: A mixed-method study into risk awareness, needs, and preferences among individuals with subjective cognitive decline and mild cognitive impairment from memory clinic and community settings and memory clinic professionals
Source: J Alzheimers Dis. 2026 Apr 17;111(3):1095–107. doi: 10.1177/13872877261440958 (PMC13219763; doi:10.1177/13872877261440958)
Supplement: sj-docx-2-alz-10.1177_13872877261440958 - Supplemental material for Towards specialized dementia risk reduction services for those with first cognitive symptoms: A mixed-method study into risk awareness, needs, and preferences among individuals with subjective cognitive decline and mild cognitive im [file sj-docx-2-alz-10.1177_13872877261440958.docx]

**Supplemental Material 2 – Topic list for interviews with individuals with SCD and MCI and memory clinic professionals**

*Topic list for interviews with individuals with SCD and MCI*

1. Can you briefly tell us about your memory complaints and why you visited the memory clinic?
2. What happened after your visit to the memory clinic?
   - How did you deal with the information you received from your doctor?
   - Did you talk to anyone about it?
   - Did you look up additional (online) information? What kind of information?

Based on our experiences at the memory clinic, we are working on developing a tool.

We want this tool to provide information about brain health and the way you live (such as what you eat or how much you exercise).

The tool is intended for people who have been to the memory clinic but do not have dementia.

1. For what purpose would you want to use this tool?

- Is it for knowledge, personalized tips, insight into your own lifestyle; or all of these?

Because we want to make the use of the tool as easy as possible, we are considering offering it online.

1. What would you think if the tool were **online**?
   - For example, a website or app on your phone?
2. Do you ever search online for information about a healthy lifestyle? Do you ever search online for information about the brain? Why or why not?
   - What do you search for? Or what have you searched for in the past month?
   - Which websites did you visit at that time?

Thinking again about the online tool we want to develop, with information about brain health and the way you live.

1. When you think of a tool about lifestyle and the brain, what do you hope to find or read in such a tool?
   - Mainly practical tips about what you can do yourself, or more background information about why you should do it?
2. Would you prefer more general information, or information tailored specifically to you? Would you like to select relevant factors yourself?
3. Would you prefer to receive **short pieces of information** (2–3 minutes reading), **or more extensive information** (a 5-minute video or 10 minutes reading)? Why?

We are also thinking about what wording to use in the tool. We are unsure how to describe the purpose of the information in the tool.

For example, we could say that the information in the tool is about:

**reducing your risk of dementia**

But we could also explain it differently, and say the **information** in the tool is to:

**stimulate your brain health**

Which wording appeals more to you?

1. What do you think is the best description of the options I just gave you? Why?
2. Do you have any other ideas for a good term we could use?

We would like to make the tool with information about your brain health and lifestyle accessible online.
So that people can use it online.

1. If you were to use the tool, on **which device** would you prefer to access it?

*For example, a computer, a phone, or a tablet*

- - Is there a particular reason for this?

🡪 You can link this to what someone said earlier. For example, someone wants extensive information, but on a phone; is that practical? What do they think?

1. If you were to use the tool, **how** **often** would you want to use it?

*For example, once a day or once a month.*

- Same as above; someone wants short pieces of information, but is once a month enough? What do they think?

1. If you use the tool once, **how long** would you want to spend on it?

*For example, 5 minutes or half an hour?*

- Same as above; someone may want short pieces of information, but still want to use it for 30 minutes?

1. Would you prefer to use the online tool **together with someone** *(for example, a partner, doctor, nurse)*? Or would you prefer to use it alone?
   - How could that person help you use the tool?
2. Would you like to also have contact with a **coach** alongside the tool?
   - How could that coach help you? And if not: why not?

*For example, filling out the first questions together, motivating you, answering your questions, etc.*

- - Could this be a **digital** coach? For example, through video calling, email, or chat?

Imagine: we have now developed the online tool with information about brain health and the way you live.
And your doctor advises you to use this tool.

1. Is there anything that would stop you from using the tool?
   - What can we do to resolve that? What would reassure you?
2. Are there maybe also things that would give you reasons or motivation to use the tool?
   - Could the coach or, for example, the doctor or nurse do something to motivate you to use the tool?

We have now discussed many things about that online tool with information about your brain health and the way you live. You have already given us many helpful tips and insights.

1. What do you now think is the most important thing to share with us regarding the tool?
2. Would you like to add anything to our conversation, or did I forget to ask something important?

**Thank you for your time!**

*Topic list for interviews with memory clinic professionals*

1. Can you briefly describe the care you provide to people with subjective or mild cognitive complaints?
2. Could you describe your typical process with someone with subjective or mild cognitive complaints?
   - How often do you typically see someone with these complaints?
   - What kind of information do you usually provide to this person?

Based on our experiences at the memory clinic, we are working on developing a tool.

We want this tool to provide information about brain health and lifestyle (for example, what someone eats or how much they exercise).

The tool is intended for people who have been to the memory clinic but do not have dementia.
The tool could possibly be used together with a healthcare provider like you, for example, to start a conversation about lifestyle and brain health.

We notice that healthcare providers often feel empty-handed when someone with subjective or mild cognitive complaints comes to them. We want to create something for that.

1. What do you think of this idea?
2. Would you want to use such a tool in practice? And if so, in what way?

Because we want to make the use of the tool as easy as possible, we are considering offering it online.

1. What would you think if the tool were **online**?
2. Do you ever refer to online information about the relationship between lifestyle and brain health? Why or why not?

Returning to the online tool we want to develop, with information about brain health and lifestyle.

1. In your opinion, what kind of information should our tool contain?
   - Mainly practical tips on what someone can do themselves, or more background information about why they should do something?
2. Would you prefer **short pieces of information** (2–3 minutes reading) in the tool, or more **extensive information** (a 5-minute video or 10 minutes reading)? Why?

We are also thinking about what wording to use in the tool. We are unsure how to describe the purpose of the information in the tool.

For example, we could say that the information in the tool is about:

**reducing your risk of dementia**

But we could also explain it differently, and say the **information** in the tool is to:

**stimulate your brain health**

Which wording appeals more to you?

1. What do you think is the best description of the options I just gave you? Why?
2. Do you have any other ideas for a good term we could use?

We would like to make the tool with information about brain health and lifestyle accessible online.

1. If you were to use the tool, on **which device** would you prefer to access it?

*For example, a computer, a phone, or a tablet*

- - Is there a particular reason for this?

🡪 You can link this to what someone said earlier. For example, someone wants extensive information, but on a phone; is that practical? What do they think?

Patients may also want to be coached in using the tool by a healthcare provider (whether or not from the memory clinic).

1. What do you think of this idea?
   - How could that coach best help the patient in your opinion?

*For example, completing the first questions together, motivating them, answering their questions, etc.*

- - Who do you think would be best suited to provide the coaching?
  - Could this be a **digital** coach? For example, through video calling, email, or chat?

Imagine: we now have that online tool with information about the relationship between lifestyle and brain health. **[EXAMPLE: DEMIJNBREINCOACH APP]** And you were to start using this tool in your practice.

1. Is there anything that would stop you from using the tool?
   - What can we do to resolve that? What would reassure you?
2. Are there maybe also things that would give you reasons or motivation to use the tool?

We have now discussed many things about that online tool with information about brain health and the way you live. You have already given us many valuable tips and insights.

1. What do you now think are the most important components of the tool?
2. Would you like to add anything to our conversation, or did I forget to ask something important?

**Thank you for your time!**
