# Supplementary material for: Towards specialized dementia risk reduction services for those with first cognitive symptoms: A mixed-method study into risk awareness, needs, and preferences among individuals with subjective cognitive decline and mild cognitive impairment from memory clinic and community settings and memory clinic professionals
Source: J Alzheimers Dis. 2026 Apr 17;111(3):1095–107. doi: 10.1177/13872877261440958 (PMC13219763; doi:10.1177/13872877261440958)
Supplement: sj-docx-3-alz-10.1177_13872877261440958 - Supplemental material for Towards specialized dementia risk reduction services for those with first cognitive symptoms: A mixed-method study into risk awareness, needs, and preferences among individuals with subjective cognitive decline and mild cognitive im [file sj-docx-3-alz-10.1177_13872877261440958.docx]

**Supplemental Material 3. Full and stratified results of awareness, needs and preferences on dementia risk reduction among surveyed participants with SCD and MCI, and memory clinic professionals**

Supplemental Table 1. Full results of awareness on dementia risk reduction among surveyed participants with SCD (n = 1,092) and MCI (n = 75)

*Answer to statement: “There is nothing you can do to lower your risk of dementia.”*

|  | Frequency | % |
| --- | --- | --- |
| Strongly agree | 18 | 1.54 |
| Agree | 103 | 8.83 |
| Neiter agree nor disagree | 340 | 29.13 |
| Disagree | 584 | 50.04 |
| Strongly disagree | 122 | 10.45 |

Supplemental Table 2. Results of awareness on dementia risk reduction among surveyed participants with SCD (n = 1,092) and MCI (n = 75) stratified by educational level

*Answer to statement: “There is nothing you can do to lower your risk of dementia.”*

|  | Low education | Middle education | High education |
| --- | --- | --- | --- |
| Disagree or doubt, n (%) | 34 (2.9) | 223 (19.1) | 204 (17.5) |
| Agree, n (%) | 30 (2.6) | 284 (24.3) | 392 (33.6) |

Supplemental Table 3. Results of awareness on dementia risk reduction among surveyed participants with SCD (n = 1,092) and MCI (n = 75) stratified by memory clinic visit

*Answer to statement: “There is nothing you can do to lower your risk of dementia.”*

|  | Visited memory clinic | Did not visit memory clinic or unsure |
| --- | --- | --- |
| Disagree or doubt, n (%) | 100 (8.6) | 361 (30.9) |
| Agree, n (%) | 137 (11.7) | 569 (48.8) |

Supplemental Table 4. Results of interest in dementia risk reduction among surveyed participants with SCD (n = 1,092) and MCI (n = 75) stratified by educational level

*Answer to statement: “Would you be interested in information about how to improve your brain health?”*

|  | Low education | Middle education | High education |
| --- | --- | --- | --- |
| Yes, n (%) | 36 (3.2) | 378 (33.8) | 472 (42.3) |
| Maybe, n (%) | 18 (1.6) | 93 (8.3) | 91 (8.2) |
| No, n (%) | 2 (0.2) | 16 (1.4) | 11 (1.0) |

Supplemental Table 5. Results of interest in dementia risk reduction among surveyed participants with SCD (n = 1,092) and MCI (n = 75) stratified by memory clinic visit

*Answer to statement: “Would you be interested in information about how to improve your brain health?”*

|  | Visited memory clinic | Did not visit memory clinic or unsure |
| --- | --- | --- |
| Yes, n (%) | 176 (15.8) | 710 (63.6) |
| Maybe, n (%) | 39 (3.5) | 163 (14.6) |
| No, n (%) | 7 (0.6) | 22 (2.0) |

Supplemental Table 6. Results of preferred source of information on dementia risk reduction among surveyed participants with SCD (n = 1,092) and MCI (n = 75) stratified by educational level

*Answer to statement: Suppose you would like to know more about your own brain health. What would you prefer?*

|  | Low education | Middle education | High education |
| --- | --- | --- | --- |
| Printed leaflet or brochure, n (%) | 6 (0.5) | 47 (4.2) | 32 (2.9) |
| Information on the internet, e.g. website, mobile app, video, n (%) | 22 (2.0) | 149 (13.3) | 227 (20.3) |
| A combination of the above-mentioned options, n (%) | 24 (2.2) | 240 (21.5) | 60 (5.4) |
| Other, n (%) | 4 (0.4) | 51 (4.6) | 60 (5.4) |

Supplemental Table 7. Results of preferred source of information on dementia risk reduction among surveyed participants with SCD (n = 1,092) and MCI (n = 75) stratified by memory clinic visit

*Answer to statement: Suppose you would like to know more about your own brain health. What would you prefer?*

|  | Visited memory clinic | Did not visit memory clinic or unsure |
| --- | --- | --- |
| Printed leaflet or brochure, n (%) | 21 (1.9) | 64 (5.7) |
| Information on the internet, e.g. website, mobile app, video, n (%) | 62 (5.6) | 336 (30.1) |
| A combination of the above-mentioned options, n (%) | 105 (9.4) | 414 (37.1) |
| Other, n (%) | 34 (3.0) | 81 (7.25) |

Supplemental Table 8. Results of awareness on dementia risk reduction among surveyed participants with SCD (n = 1,092) and MCI (n = 75) stratified by educational level

*Answer to statement: “Suppose a website or app (mobile application) was developed that gives you free information about your brain health and how to improve it. Would you want to use it?”*

|  | Low education | Middle education | High education |
| --- | --- | --- | --- |
| Yes, n (%) | 45 (4.0) | 391 (35.0) | 471 (42.2) |
| Maybe, n (%) | 11 (1.0) | 87 (1.8) | 88 (7.9) |
| No, n (%) | 0 (0) | 9 (0.8) | 15 (1.3) |

Supplemental Table 9. Results of awareness on dementia risk reduction among surveyed participants with SCD (n = 1,092) and MCI (n = 75) stratified by memory clinic visit

*Answer to statement: “Suppose a website or app (mobile application) was developed that gives you free information about your brain health and how to improve it. Would you want to use it?”*

|  | Visited memory clinic | Did not visit memory clinic or unsure |
| --- | --- | --- |
| Yes, n (%) | 182 (16.3) | 725 (64.9) |
| Maybe, n (%) | 32 (2.9) | 154 (13.8) |
| No, n (%) | 8 (0.7) | 16 (1.4) |

Supplemental Table 10. Full results of awareness on dementia risk reduction among surveyed memory clinic professionals (n = 58)

*Answer to statement: “There is nothing you can do to lower your risk of dementia.”*

|  | Frequency | % |
| --- | --- | --- |
| Strongly agree | 0 | 0 |
| Agree | 3 | 5.17 |
| Neiter agree nor disagree | 3 | 5.17 |
| Disagree | 39 | 67.24 |
| Strongly disagree | 13 | 22.41 |
